# Impact of Beta-Lactam Target Attainment on Resistance Development in Patients with Gram-Negative Infections

**DOI:** 10.3390/antibiotics12121696

**Published:** 2023-12-03

**Authors:** Nicole F. Maranchick, Jessica Webber, Mohammad H. Alshaer, Timothy W. Felton, Charles A. Peloquin

**Affiliations:** 1 Infectious Disease Pharmacokinetics Lab, Department of Pharmacotherapy and Translational Research, College of Pharmacy, University of Florida, Gainesville, FL 32610, USA; 2 Emerging Pathogens Institute, University of Florida, Gainesville, FL 32610, USA; 3 College of Pharmacy, University of Florida, Gainesville, FL 32610, USA; 4 North West Ventilation Unit, Manchester University NHS Foundation Trust, Manchester M23 9LT, UK; 5 Division of Infection, Immunity and Respiratory Medicine, School of Biological Sciences, Faculty of Biology, Medicine and Health, The University of Manchester, Manchester M13 9NT, UK

**Keywords:** drug resistance, gram-negative bacteria, beta-lactams, pharmacokinetic/pharmacodynamic

## Abstract

Background: The objective was to identify associations between beta-lactam pharmacokinetic/pharmacodynamic (PK/PD) targets and Gram-negative bacteria resistance emergence in patients. Methods: Retrospective data were collected between 2016 to 2019 at the University of Florida Health-Shands Hospital in Gainesville, FL. Adult patients with two Gram-negative isolates receiving cefepime, meropenem, or piperacillin-tazobactam and who had plasma beta-lactam concentrations were included. Beta-lactam exposures and time free drug concentrations that exceeded minimum inhibitory concentrations (ƒT > MIC), four multiples of MIC (ƒT > 4× MIC), and free area under the time concentration curve to MIC (ƒAUC/MIC) were generated. Resistance emergence was defined as any increase in MIC or two-fold increase in MIC. Multiple regression analysis assessed the PK/PD parameter impact on resistance emergence. Results: Two hundred fifty-six patients with 628 isolates were included. The median age was 58 years, and 59% were males. Cefepime was the most common beta-lactam (65%) and *Pseudomonas aeruginosa* the most common isolate (43%). The mean daily ƒAUC/MIC ≥ 494 was associated with any increase in MIC (*p* = 0.002) and two-fold increase in MIC (*p* = 0.004). The daily ƒAUC/MIC ≥ 494 was associated with decreased time on antibiotics (*p* = 0.008). *P. aeruginosa* was associated with any increase in MIC (OR: 6.41, 95% CI [3.34–12.28]) or 2× increase in MIC (7.08, 95% CI [3.56–14.07]). Conclusions: ƒAUC/MIC ≥ 494 may be associated with decreased Gram-negative resistance emergence.

## 1. Introduction

Antimicrobial resistance puts millions of lives at risk, and in the United States, antimicrobial-resistant bacteria and fungi contribute to more than 35,000 deaths each year [1,2]. Antimicrobial resistance occurs when changes in bacteria cause the drugs used to treat them to become less effective [3]. Gram-negative resistance is especially a concern regarding Enterobacterales and *Pseudomonas aeruginosa*, which have demonstrated resistance to all available antibiotics through varying mechanisms [1,4]. Gram-negative bacteria are highly adaptable to antibiotics, warranting a judicious use to minimize resistance emergence [5]. In addition, given the slow development of new antibiotics, alternative strategies to minimize resistance emergence are necessary, such as optimizing pharmacokinetic/pharmacodynamic (PK/PD) drug targets [6,7,8,9].

Beta-lactams are the most commonly used class of antibiotics, and their bacterial killing depends upon the percent of time that free drug concentrations remain above the bacteria minimum inhibitory concentration (ƒT > MIC) [10]. Clinical data have suggested that PK/PD targets such as 100% ƒT > 4–5× MIC may be necessary to improve clinical and microbiological outcomes [11,12,13]. Given that beta-lactams are widely used due to their broad efficacy and excellent safety profile, resistance to them is particularly concerning [6]. Consensus is currently lacking as to what PK/PD target maximizes beta-lactams’ clinical efficacy while also minimizing resistance emergence [14]. It may be expected that the pharmacodynamic driver for resistance suppression would be the same as for bacterial cell killing, but this is not always the case [15]. Given the lack of evidence, the purpose of this study was to identify associations between beta-lactam PK/PD targets and resistance emergence in patients with Gram-negative bacterial infections.

## 2. Results

In total, 256 patients with 628 Gram-negative isolates were included. The median (interquartile range) age and body mass index (BMI) were 58 (42–69) years and 26 (21.4–33.4) kg/m^2^, respectively (Table 1). Seventy-nine percent of patients were critically ill at the start of beta-lactam antibiotics. The most common Gram-negative isolates were *P. aeruginosa* (43%), *Escherichia coli* (14%), and *Klebsiella pneumoniae* (8%). The most common culture types were from the lung (38%) and blood (38%). Multi-drug resistance was identified in 9.8% of isolates. Most patients received cefepime (65%). Fifteen (6%) patients received more than one study antibiotic, but not concurrently.

**Figure 1:**
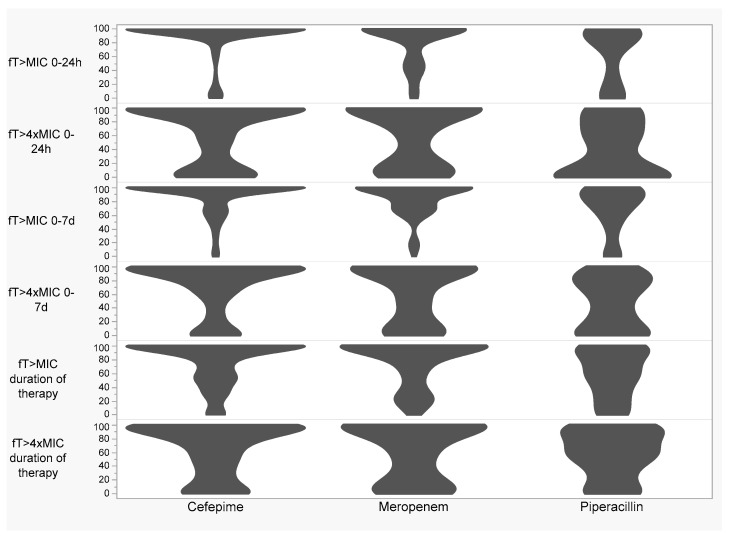
Violin plots showing PK/PD target attainment stratified by beta-lactam. PK/PD—pharmacokinetic/pharmacodynamic; MIC—minimum inhibitory concentration; ƒT > MIC—time free drug concentrations exceed minimum inhibitory concentration; ƒT > 4× MIC—time free drug concentrations exceed four multiples of MIC.

**Figure 2:**
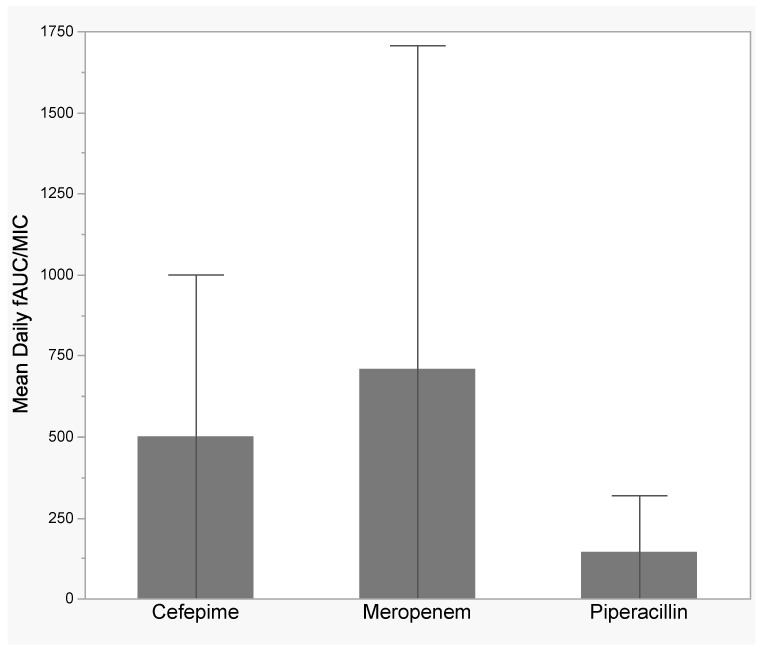
Bar graphs showing mean (standard deviation) daily ƒAUC/MIC stratified by beta-lactam. ƒAUC/MIC—free area under the time concentration curve to minimum inhibitory concentration.

Figure 1 shows beta-lactam target attainment stratified by beta-lactam and time window (0–24 h, 0–7 days, and duration of therapy). In the first 24 h, 81% and 45% (cefepime), 70% and 48% (meropenem), and 37% and 16% (piperacillin) achieved 100% ƒT > MIC and 100% ƒT > 4× MIC, respectively. In the first 7 days, 61% and 34% (cefepime), 55% and 38% (meropenem), and 18% and 2% (piperacillin) achieved 100% ƒT > MIC and 100% ƒT > 4× MIC. For the total duration of antibiotics, 49% and 24% (cefepime), 50% and 34% (meropenem), and 14% and 2% (piperacillin) achieved 100% ƒT > MIC and 100% ƒT > 4× MIC, respectively. Figure 2 shows the mean (SD) daily ƒAUC/MIC stratified by beta-lactam. Meropenem had the highest mean free area under the time concentration curve to MIC (ƒAUC/MIC) of 708 (999), followed by cefepime with a mean daily ƒAUC/MIC of 500 (499), and then piperacillin with a mean AUC/MIC of 145 (175).

Nineteen percent of isolates had an increase in MIC between cultures, of which 77% was due to *P. aeruginosa*. After testing for associations between baseline characteristics and resistance, renal replacement therapy (RRT) during admission, intensive care unit (ICU) length of stay (LOS), hospital LOS, mechanical ventilation, and days between first and second culture were significantly associated with resistance (Table 2). The Sequential Organ Failure Assessment (SOFA) score was associated with a two-fold increase in MIC. These covariates were controlled for in the multiple regression analysis (Table 3). Classification and regression tree (CART) analysis determined a significant ƒAUC/MIC target of 494 associated with resistance emergence that was included in the models. Subjects with a mean daily ƒAUC/MIC ≥ 494 had significantly less resistance emergence when defined as any increase in MIC (aOR 0.25, 95% CI [0.11–0.61]) or at least a two-fold increase in MIC (aOR 0.27, 95% CI [0.11–0.67]). These relationships held true even in a subgroup analysis including only initial and final culture types of the same source. In the subgroup analysis, a daily ƒAUC/MIC ≥ 494 was significantly associated with an increased risk of Gram-negative resistance emergence when defined as any increase in MIC (aOR 0.33, 95% CI [0.13–0.87]) or at least a two-fold increase in MIC (aOR 0.38, 95% CI [0.14–0.99]). No associations were found between ƒT > MIC and Gram-negative resistance emergence.

*P. aeruginosa* was found to be significantly associated with bacterial resistance emergence for both any increase in MIC (OR: 6.41, 95% CI [3.34–12.28]) and at least a two-fold increase in MIC (7.08, 95% CI [3.56–14.07]).

**Figure 3:**
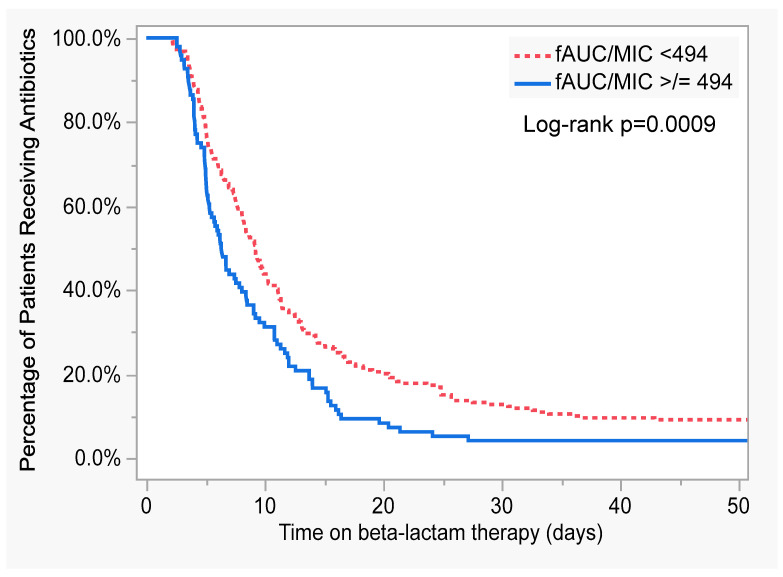
Kaplan–Meier curve for beta-lactam duration of therapy comparing patients based on ƒAUC/MIC target attainment of 494.

Figure 3 shows the Kaplan–Meier curve for the time on beta-lactams based upon the mean daily ƒAUC/MIC target attainment. Thirty-one percent achieved a mean daily ƒAUC/MIC ≥ 494. Patients achieving ƒAUC/MIC ≥ 494 had a significantly shorter time on antibiotics (*p* = 0.008).

## 3. Discussion

This study’s objective was to identify associations between beta-lactam target attainment and the prevention of resistance emergence in patients with Gram-negative infections. In the covariate analysis, RRT, mechanical ventilation, ICU LOS, hospital LOS, and days between first and last culture were associated with resistance. The SOFA score was associated with a two-fold increase in MIC. The mean daily ƒAUC/MIC ≥ 494 was associated with a decreased risk of resistance development. Time-to-event analysis showed that patients achieving a mean daily ƒAUC/MIC ≥ 494 had less time on beta-lactam therapy. These results indicate that optimizing daily beta-lactam ƒAUC/MIC exposure may minimize Gram-negative resistance and the duration of therapy.

In a previous study of 76 patients receiving either cefepime or ceftazidime, researchers aimed to characterize the relationship between PD parameters and clinical and microbiological outcomes. Patients were included if they had sepsis and suspected or proven infection due to a pathogen susceptible to cefepime/ceftazidime. Patient PK parameters were estimated from doses administered and patient-specific data. Patients achieving ƒAUC/MIC ratios ≥ 250 had significantly higher rates of clinical cure (*p* = 0.002) and bacteriological eradication (*p* < 0.001) [16]. In a study by Schentag et al., researchers performed simulations to evaluate ƒAUC/MIC targets for cefmenoxime, tobramycin, and ciprofloxacin. They found that for cefmenoxime, a cephalosporin, an ƒAUC/MIC ratio of 540 per 24 h was required for bacterial eradication. They also proposed that each antibiotic has a unique 24-h ƒAUC/MIC value associated with bacterial eradication at 4 days [17]. Our PK/PD target of 494 falls within the ranges from the previously published literature and may be beneficial for clinical outcomes, bacterial eradication, and/or resistance suppression.

In general, the efficacy of beta-lactams depends upon ƒT > MIC, with 40–70% ƒT > MIC proposed as the minimum threshold for bactericidal activity [18]. In a study by Gatti et al., 116 ICU patients receiving beta-lactam continuous infusions for Gram-negative infections with at least one therapeutic drug monitoring in the first 72 h of treatment were assessed for PK/PD target thresholds. Steady state concentration/MIC ratios ≤ 5 were associated with microbiological failure. In addition, *P. aeruginosa* infection was associated with microbiological failure [14]. Felton et al. compared piperacillin/tazobactam PK/PD indices to suppress bacterial resistance in both high and low burdens of *P. aeruginosa*. A Cmin/MIC of 3.4 was required, unless the bacterial burden was high, in which case a Cmin/MIC of 4.6 was needed [19]. In our study, we did not find an association between ƒT > MIC and ƒT > 4× MIC with the emergence of Gram-negative resistance. However, based upon the findings of the previous study and increased target concentrations with a high bacterial burden, there is the potential that our targets of ƒT > MIC and ƒT > 4× MIC were not sufficient to suppress bacterial resistance. In addition, *P. aeruginosa* was responsible for approximately 43% of the isolates in our study and was an independent risk factor for resistance emergence, which may have impacted the ability of PK/PD target attainment to suppress resistance emergence.

Beta-lactam target attainment (ƒT > MIC, ƒT > 4× MIC, and ƒAUC/MIC ≥ 494) for patients in the present study was in general poor, especially for piperacillin. The low target attainment could be due to the high percentage of ICU patients (approximately 79%) who have an increased risk of pharmacokinetic variability. Of note, while patients achieving daily ƒAUC/MIC ≥ 494 had significantly less time on antibiotics, only 31% of patients met this target. In a previous study of 80 ICU patients receiving cefepime, meropenem, and piperacillin-tazobactam, researchers found that serum concentrations remained 4× above target concentrations for the *P. aeruginosa* breakpoint for 34% (cefepime), 57% (meropenem), and 33% (piperacillin-tazobactam) of the dosing interval. They concluded that only meropenem had acceptable serum concentrations and that more aggressive dosing may be needed to empirically cover pathogens, especially in critically ill patients [20]. The EXPAT study was a prospective observational study in two ICUs. Researchers enrolled patients receiving beta-lactam antibiotics and collected drug samples on day 2 of therapy. Of 147 patients, researchers concluded that 63.3% and 36.7% of patients achieved 100% ƒT > MIC and 100% ƒT > 4× MIC, respectively. They identified male gender, high BMI, and elevated eGFR as risk factors for target non-attainment [21]. While our study did not find an association between ƒT > MIC or ƒT > 4× MIC, we have demonstrated that ƒAUC/MIC target attainment decreased time on antibiotics, which provides further support for early target attainment in clinical practice. Therefore, due to the risks of subtherapeutic beta-lactam concentrations and the benefits of early target attainment, especially in critically ill patients, therapeutic drug monitoring would ideally be initiated on day 1 of beta-lactam therapy to improve target attainment.

There are limitations to this study. This was a retrospective study utilizing data from a single center with a high percentage of critically ill patients, which may limit external generalizability to other sites. In addition, protein binding values were estimated and used to calculate the unbound fraction, as total drug concentrations were measured. Unbound fractions may be highly variable due to critical illness, hypoalbuminemia, and chronic kidney disease. Sixteen percent of patients received renal replacement therapy during beta-lactam therapy. However, the models used to generate beta-lactam exposures do not account for patients receiving renal replacement therapy, so beta-lactam exposures may have been impacted. In addition, it is generally accepted that MICs have variability, with repeat assessments potentially producing MIC values that differ by 200% [22]. Additionally, due to the retrospective nature of the study, bacterial cultures were ordered by the treating team and were not taken at scheduled intervals. Although controlled for in analysis, future studies should utilize scheduled intervals to obtain cultures to assess resistance emergence.

## 4. Materials and Methods

### 4.1. Data Collection

This retrospective study utilized pooled data from the UF Health-Shands hospital in Gainesville, Florida, USA between 2016 to 2019. Patients were included if ≥18 years old, had two separate cultures positive for Gram-negative bacteria during the same hospitalization, were receiving cefepime, meropenem, or piperacillin (administered with tazobactam), and had beta-lactam concentrations measured during therapy as part of the usual therapeutic drug monitoring service [23]. Cultures were included if collected from the same site at least one day apart. The first culture was included if it was before or at the start of antibiotic therapy, and subsequent cultures were included if collected up to 30 days after therapy discontinuation. Additional data collected included age, BMI, ICU LOS, hospital LOS, SOFA score, days on antibiotic therapy, days between cultures, and clinical factors, such as renal replacement therapy, mechanical ventilation, diabetes, liver disease, chronic obstructive pulmonary disease (COPD), and heart failure. These covariates were chosen due to their potential to be associated with resistance emergence [24,25,26,27].

Resistance was defined as any increase in minimum inhibitory concentration (MIC) or at least a two-fold increase in MIC. If the second culture had no growth, it was considered susceptible. If multiple MICs were available after the first culture, the highest available MIC was used. MICs were determined by the UF-Health Shands microbiology. Methods to identify bacteria and MICs included VITEK^®^ Mass Spectrometry and Vitek^®^ II (bioMérieux, Inc., Durham, NC, USA). Etest was utilized for MIC quantification for the following bacteria (beta-lactam) combinations: *Acinetobacter* spp. (cefepime, meropenem, and piperacillin), *Burkholderia cepacia complex* (meropenem), and Gram-negative non-fermenters (cefepime, meropenem, and piperacillin). Multidrug-resistant (MDR) isolates were defined as extended spectrum beta-lactamase (ESBL) *Enterobacterales*, carbapenem-resistant (CR) *Enterobacterales*, MDR-*Pseudomonas aeruginosa*, MDR-*Acinetobacter baumannii*, or any bacteria non-susceptible to ≥1 agent in ≥3 antimicrobial categories [4,28,29,30].

Following collection, total beta-lactam plasma concentrations were quantified using liquid chromatography with tandem mass spectrometry assays at the Infectious Disease Pharmacokinetics Laboratory at UF. The calibration range was 2–100 mg/L with an inter- and intra-day accuracy and precision < 10%. Values below the limit of quantification were assigned a value of “0” for analysis. Free drug concentrations were estimated using previously published values (80% for cefepime, 98% for meropenem, and 70% for piperacillin) [31,32,33].

Posterior predictions were generated using the nonparametric adaptive grid (NPAG) in Pmetrics v1.9.7 (Laboratory of Applied Pharmacokinetics and Bioinformatics, Los Angeles, CA, USA) with previously published cefepime, meropenem, and piperacillin models, drug doses, drug concentrations, and covariates, including renal function (CrCl or SCr), weight, and age [34,35,36]. Beta-lactam exposure was generated from initiation of antibiotics up until therapy discontinuation or the time of resistant bacteria culture collection, whichever was reported first. Beta-lactam posterior predictions were imported to Phoenix WinNonlin v8.3.4 (Certara, Princeton, NJ, USA) to calculate the free area under the time concentration curve (ƒAUC) and ƒT > MIC and ƒT > 4× MIC for 0–24 h, 0–7 days, and the duration of beta-lactam therapy or up to the day of resistant bacterial culture. Calculated ƒAUC was used to estimate mean daily ƒAUC to MIC (ƒAUC/MIC) ratios. If patients received beta-lactam therapy for less than or had a resistant bacteria culture in less than 7 days, PK/PD calculations were stopped after the last dose of beta-lactam.

### 4.2. Statistical Analysis

Statistical analysis was performed on JMP Pro v17 (SAS Institute, Cary, NC, USA). Continuous data were presented as the median and interquartile range (IQR) and categorical data as count and percentages. Covariates including age, BMI, RRT, days on antibiotics, days between first and last culture, mechanical ventilation, hospital and ICU LOS, diabetes, liver disease, COPD, heart failure, and SOFA score were tested individually for association with resistance emergence (defined as both any increase in MIC or ≥2× increase in MIC) in a univariate analysis. Significant covariates identified in the univariate analysis were included and controlled for in the final multiple regression models, and PK/PD parameters were tested individually for associations with bacterial resistance. PK/PD parameters tested in the multiple regression models included the mean daily ƒAUC/MIC and ƒT > MIC and ƒT > 4× MIC for 0 to 24 h, 0 to 7 days, and the duration of therapy. Classification and regression tree (CART) analysis was used to test PK/PD parameters including fT > MIC and ƒAUC/MIC for breakpoint values of significance. These breakpoints were then tested in the multiple regression models for associations with bacterial resistance. A subgroup analysis was also conducted to test PK/PD relationships with resistance emergence when only using cultures of the same source. The same significant covariates were included in the final multiple regression model. Kaplan–Meier estimators were reported for associations between PK/PD parameters and the time on beta-lactam therapy. A *p*-value of less than 0.05 was considered statistically significant for all analyses.

## 5. Conclusions

Daily ƒAUC/MIC ≥ 494 may be associated with a decreased risk of Gram-negative resistance emergence and a reduced duration of antibiotics. These results could be a potential PK/PD target for future investigations, such as interventional prospective studies with a larger sample size. *P. aeruginosa* was significantly associated with an increase in resistance emergence. No associations between resistance emergence and ƒT > MIC and ƒT > 4× MIC were found.

## Figures and Tables

**Table 1:** Patient characteristics, *n* = 256 patients.

Clinical Characteristics	Median (IQR) or *n* (%)
Age, years	58 (42–69)
Sex, Male	151 (59)
Weight, kg	73.6 (60.9–94.3)
Serum creatinine, mg/dL	0.81 (0.57–1.27)
BMI, kg/m^2^	26 (21.4–33.4)
Renal replacement therapy	44 (17)
Liver Disease	78 (30)
COPD	55 (21)
Diabetes	113 (44)
Heart failure	105 (41)
Mechanical Ventilation	66 (26)
Patient in ICU at beta-lactam initiation	203 (79)
Length of stay, days ICU Hospital	14 (2–29) 25 (16–47)
Mortality	122 (48)
Gram-negative isolates, *n*	628
Common isolated bacteria, *n* (% developing resistance) *Pseudomonas aeruginosa* *Escherichia coli* *Klebsiella pneumoniae* *Enterobacter cloacae* *Serratia marcescens* *Proteus mirabilis* *Acinetobacter baumannii* *Klebsiella aerogenes*	134 (35.6) 30 (6.7) 26 (11.5) 20 (0) 19 (5.2) 14 (0) 14 (14.2) 10 (10)
Multi-drug resistant isolates ^#^	62 (9.8)
All Culture Sources Lung Blood Wound Urinary Tract Abscess/body fluid Other	233 230 80 36 32 17
Culture Sources (Same Final and Initial Source) Lung Blood Wound Urinary Tract Abscess/body fluid Other	204 214 46 32 16 6
Beta-lactam Received ^^^ Cefepime Meropenem Piperacillin/tazobactam	179 (65) 54 (20) 41 (15)
Number of samples Cefepime Meropenem Piperacillin/tazobactam	316 91 73
Beta-lactam therapy duration, days	8 (5–14)
Time between cultures, days	7 (4–15)
Time between start of beta-lactam therapy and TDM, days	3 (2–8)
Concomitant Antibiotics -Aminoglycoside -Fluoroquinolone -Polymyxin	124 (48) 57 (22) 30 (12)

IQR—interquartile range; BMI—body mass index; COPD—chronic obstructive pulmonary disease; ICU—intensive care unit; TDM—therapeutic drug monitoring; ^ Total = 274. Fifteen patients received more than one beta-lactam during the study period; ^#^ Multidrug resistant (MDR) isolates include extended spectrum beta-lactamase (ESBL) *Enterobacterales*, carbapenem-resistant (CR) *Enterobacterales*, MDR-*Pseudomonas aeruginosa*, MDR-*Acinetobacter baumannii*, or any bacteria non-susceptible to ≥1 agent in ≥3 antimicrobial categories.

**Table 2:** Univariate analysis.

	Resistance (Any Increase in MIC)		Resistance (≥2× MIC Increase)	
Covariates	OR	*p*-Value	OR	*p*-Value
Age (per 1 year)	0.99	0.08	0.99	0.35
BMI (per 1 kg/m^2^)	1.00	0.73	1.00	0.82
RRT during admission	2.24	**0.03**	2.50	**0.01**
Days on antibiotic therapy (per 1 day)	1.01	0.06	1.01	0.06
Days between cultures (per 1 day)	1.06	**<0.0001**	1.06	**<0.0001**
Mechanical Ventilation	2.27	**0.007**	2.61	**0.002**
Hospital LOS (per 1 day)	1.01	**<0.0001**	1.01	**<0.0001**
ICU LOS (per 1 day)	1.02	**<0.0001**	1.02	**<0.0001**
Diabetes	1.14	0.67	0.94	0.88
Liver Disease	1.38	0.28	1.30	0.43
COPD	1.38	0.39	1.38	0.38
Heart Failure	1.18	0.56	1.36	0.30
SOFA Score (per 1 point)	1.05	0.12	1.08	**0.02**

Note: MIC—minimum inhibitory concentration; BMI—body mass index; RRT—renal replacement therapy; LOS—length of stay; ICU—intensive care unit; COPD—chronic obstructive pulmonary disease; SOFA—sequential organ failure assessment.

**Table 3:** Final statistical models with PK/PD predictors.

	Resistance (Any Increase in MIC)		Resistance (≥2× MIC Increase)	
PK/PD Parameter	aOR (95% CI)	*p*-Value	aOR (95% CI)	*p*-Value
% ƒT > MIC 0–24 h (per 10%)	0.96 (0.88–1.07)	0.50	1.06 (0.95–1.20)	0.34
% ƒT > 4× MIC 0–24 h (per 10%)	0.98 (0.91–1.06)	0.64	1.02 (0.94–1.10)	0.71
% ƒT > MIC 0–7 d (per 10%)	1.06 (0.95–1.21)	0.31	1.08 (0.94–1.24)	0.27
% ƒT > 4× MIC 0–7 d (per 10%)	1.03 (0.94–1.12)	0.51	1.08 (0.99–1.19)	0.08
% ƒT > MIC duration of therapy (per 10%)	0.98 (0.89–1.08)	0.67	1.03 (0.92–1.14)	0.63
% ƒT > 4× MIC duration of therapy (per 10%)	0.99 (0.92–1.09)	0.95	1.03 (0.94–1.13)	0.53
Mean daily ƒAUC/MIC (per increments of 10)	0.99 (0.98–1.00)	0.08	1.00 (0.99–1.001)	0.17
Mean daily ƒAUC/MIC of 494 achieved (yes)	0.25 (0.11–0.61)	**0.002**	0.27 (0.11–0.67)	**0.004**

Note: MIC—minimum inhibitory concentration; PK/PD—pharmacokinetic/pharmacodynamic; aOR—adjusted odds ratio; CI—confidence interval.

## Data Availability

Data supporting the reported results are available upon request from the corresponding author.

## References

[1] Centers for Disease Control and Prevention (2022). About Antimicrobial Resistance. https://www.cdc.gov/drugresistance/about.html.

[2] World Health Organization Antimicrobial Resistance: Global Report on Surveillance. 1 April 2014. https://www.who.int/publications/i/item/9789241564748.

[3] Murray C.J.L., Ikuta K.S., Sharara F., Swetschinski L., Aguilar G.R., Gray A., Han C., Bisignano C., Rao P., Wool E. (2022). Global burden of bacterial antimicrobial resistance in 2019: A systematic analysis. Lancet.

[4] Magiorakos A.-P., Srinivasan A., Carey R.B., Carmeli Y., Falagas M.E., Giske C.G., Harbarth S., Hindler J.F., Kahlmeter G., Olsson-Liljequist B. (2012). Multidrug-resistant, extensively drug-resistant and pandrug-resistant bacteria: An international expert proposal for interim standard definitions for acquired resistance. Clin. Microbiol. Infect..

[5] Fernandes P. (2006). Antibacterial discovery and development—The failure of success?. Nat. Biotechnol..

[6] MacVane S.H. (2016). Antimicrobial Resistance in the Intensive Care Unit: A Focus on Gram-Negative Bacterial Infections. J. Intensiv. Care Med..

[7] Sumi C.D., Heffernan A.J., Lipman J., Roberts J.A., Sime F.B. (2019). What Antibiotic Exposures Are Required to Suppress the Emergence of Resistance for Gram-Negative Bacteria? A Systematic Review. Clin. Pharmacokinet..

[8] Heffernan A.J., Sime F.B., Lipman J., Roberts J.A. (2018). Individualising Therapy to Minimize Bacterial Multidrug Resistance. Drugs.

[9] Adembri C., Novelli A., Nobili S. (2020). Some Suggestions from PK/PD Principles to Contain Resistance in the Clinical Setting—Focus on ICU Patients and Gram-Negative Strains. Antibiotics.

[10] Rybak M.J. (2006). Pharmacodynamics: Relation to antimicrobial resistance. Am. J. Infect. Control.

[11] Alshaer M.H., Maranchick N., Bai C., Maguigan K.L., Shoulders B., Felton T.W., Mathew S.K., Mardini M.T., Peloquin C.A. (2022). Using Machine Learning to Define the Impact of Beta-Lactam Early and Cumulative Target Attainment on Outcomes in Intensive Care Unit Patients with Hospital-Acquired and Ventilator-Associated Pneumonia. Antimicrob. Agents Chemother..

[12] Roberts J.A., Paul S.K., Akova M., Bassetti M., De Waele J.J., Dimopoulos G., Kaukonen K.-M., Koulenti D., Martin C., Montravers P. (2014). DALI: Defining Antibiotic Levels in Intensive Care Unit Patients: Are Current β-Lactam Antibiotic Doses Sufficient for Critically Ill Patients?. Clin. Infect. Dis..

[13] Guilhaumou R., Benaboud S., Bennis Y., Dahyot-Fizelier C., Dailly E., Gandia P., Goutelle S., Lefeuvre S., Mongardon N., Roger C. (2019). Optimization of the treatment with beta-lactam antibiotics in critically ill patients—Guidelines from the French Society of Pharmacology and Therapeutics (Société Française de Pharmacologie et Thérapeutique—SFPT) and the French Society of Anaesthesia and Intensive Care Medicine (Société Française d’Anesthésie et Réanimation—SFAR). Crit. Care.

[14] Gatti M., Cojutti P.G., Pascale R., Tonetti T., Laici C., Dell’olio A., Siniscalchi A., Giannella M., Viale P., Pea F. (2021). Assessment of a PK/PD Target of Continuous Infusion Beta-Lactams Useful for Preventing Microbiological Failure and/or Resistance Development in Critically Ill Patients Affected by Documented Gram-Negative Infections. Antibiotics.

[15] Drusano G.L., Louie A., MacGowan A., Hope W. (2016). Suppression of Emergence of Resistance in Pathogenic Bacteria: Keeping Our Powder Dry, Part 1. Antimicrob. Agents Chemother..

[16] McKinnon P.S., Paladino J.A., Schentag J.J. (2008). Evaluation of area under the inhibitory curve (AUIC) and time above the minimum inhibitory concentration (T>MIC) as predictors of outcome for cefepime and ceftazidime in serious bacterial infections. Int. J. Antimicrob. Agents.

[17] Schentag J.J., Nix D.E., Adelman M.H. (1991). Mathematical Examination of Dual Individualization Principles (I): Relationships between AUC above MIC and Area under the Inhibitory Curve for Cefmenoxime, Ciprofloxacin, and Tobramycin. DICP.

[18] Craig W.A. (1998). State-of-the-Art Clinical Article: Pharmacokinetic/Pharmacodynamic Parameters: Rationale for Antibacterial Dosing of Mice and Men. Clin. Infect. Dis..

[19] Felton T.W., Goodwin J., O’Connor L., Sharp A., Gregson L., Livermore J., Howard S.J., Neely M.N., Hope W.W. (2013). Impact of Bolus Dosing versus Continuous Infusion of Piperacillin and Tazobactam on the Development of Antimicrobial Resistance in Pseudomonas aeruginosa. Antimicrob. Agents Chemother..

[20] Taccone F.S., Laterre P.-F., Dugernier T., Spapen H., Delattre I., Witebolle X., De Backer D., Layeux B., Wallemacq P., Vincent J.-L. (2010). Insufficient β-lactam concentrations in the early phase of severe sepsis and septic shock. Crit. Care.

[21] Abdulla A., Dijkstra A., Hunfeld N.G.M., Endeman H., Bahmany S., Ewoldt T.M.J., Muller A.E., van Gelder T., Gommers D., Koch B.C.P. (2020). Failure of target attainment of beta-lactam antibiotics in critically ill patients and associated risk factors: A two-center prospective study (EXPAT). Crit. Care.

[22] Doern G.V., Brecher S.M. (2011). The Clinical Predictive Value (or Lack Thereof) of the Results of In Vitro Antimicrobial Susceptibility Tests. J. Clin. Microbiol..

[23] Venugopalan V., Hamza M., Santevecchi B., DeSear K., Cherabuddi K., Peloquin C.A., Alshaer M.H. (2022). Implementation of a β-lactam therapeutic drug monitoring program: Experience from a large academic medical center. Am. J. Health Pharm..

[24] Hayakawa K., Gattu S., Marchaim D., Bhargava A., Palla M., Alshabani K., Gudur U.M., Pulluru H., Bathina P., Sundaragiri P.R. (2013). Epidemiology and Risk Factors for Isolation of Escherichia coli Producing CTX-M-Type Extended-Spectrum β-Lactamase in a Large U.S. Medical Center. Antimicrob. Agents Chemother..

[25] Ben-Ami R., Rodríguez-Baño J., Arslan H., Pitout J.D.D., Quentin C., Calbo E.S., Azap K., Arpin C., Pascual A., Livermore D.M. (2009). A Multinational Survey of Risk Factors for Infection with Extended-Spectrum β-Lactamase–Producing Enterobacteriaceae in Nonhospitalized Patients. Clin. Infect. Dis..

[26] Maina J.W., Onyambu F.G., Kibet P.S., Musyoki A.M. (2023). Multidrug-resistant Gram-negative bacterial infections and associated factors in a Kenyan intensive care unit: A cross-sectional study. Ann. Clin. Microbiol. Antimicrob..

[27] Al Hamdan A.S., Alghamdi A., Alyousif G.F., Hamza F., Shafey M.M., AlAmri A.M., Sunki A.A. (2022). Evaluating the Prevalence and the Risk Factors of Gram-Negative Multi-Drug Resistant Bacteria in Eastern Saudi Arabia. Infect. Drug Resist..

[28] Tamma P.D., Aitken S.L., Bonomo R.A., Mathers A.J., van Duin D., Clancy C.J. (2023). Infectious Diseases Society of America 2023 Guidance on the Treatment of Antimicrobial Resistant Gram-Negative Infections. Clin. Infect. Dis..

[29] Wang M., Wei H., Zhao Y., Shang L., Di L., Lyu C., Liu J. (2019). Analysis of multidrug-resistant bacteria in 3223 patients with hospital-acquired infections (HAI) from a tertiary general hospital in China. Bosn. J. Basic Med. Sci..

[30] Balkhair A., Al-Farsi Y.M., Al-Muharrmi Z., Al-Rashdi R., Al-Jabri M., Neilson F., Al-Adawi S.S., El-Beeli M., Al-Adawi S. (2014). Epidemiology of Multi-Drug Resistant Organisms in a Teaching Hospital in Oman: A One-Year Hospital-Based Study. Sci. World J..

[31] Wong G., Briscoe S., Adnan S., McWhinney B., Ungerer J., Lipman J., Roberts J.A. (2013). Protein Binding of β-Lactam Antibiotics in Critically Ill Patients: Can We Successfully Predict Unbound Concentrations?. Antimicrob. Agents Chemother..

[32] Craig W.A. (1997). The Pharmacology of Meropenem, A New Carbapenem Antibiotic. Clin. Infect. Dis..

[33] Adnan S., Paterson D.L., Lipman J., Kumar S., Li J., Rudd M., Roberts J.A. (2012). Pharmacokinetics of Beta-Lactam Antibiotics in Patients with Intra-Abdominal Disease: A Structured Review. Surg. Infect..

[34] Alshaer M.H., Goutelle S., Santevecchi B.A., Shoulders B.R., Venugopalan V., Cherabuddi K., Liu J., Kiel P.J., Roberts J.A., Sime F.B. (2022). Cefepime Precision Dosing Tool: From Standard to Precise Dose Using Nonparametric Population Pharmacokinetics. Antimicrob. Agents Chemother..

[35] Mathew S.K., Mathew B.S., Neely M.N., Naik G.S., Prabha R., Jacob G.G., Subramani K., Fleming D.H. (2016). A Nonparametric Pharmacokinetic Approach to Determine the Optimal Dosing Regimen for 30-Minute and 3-Hour Meropenem Infusions in Critically Ill Patients. Ther. Drug Monit..

[36] Felton T.W., Roberts J.A., Lodise T.P., Van Guilder M., Boselli E., Neely M.N., Hope W.W. (2014). Individualization of Piperacillin Dosing for Critically Ill Patients: Dosing Software to Optimize Antimicrobial Therapy. Antimicrob. Agents Chemother..

